# GLFNet: Combining Global and Local Information in Vehicle Re-Recognition

**DOI:** 10.3390/s24020616

**Published:** 2024-01-18

**Authors:** Yinghan Yang, Peng Liu, Junran Huang, Hongfei Song

**Affiliations:** 1 College of Automotive Engineering, Jilin University, Changchun 130012, China; 13756598231@163.com; 2 School of Mechanical and Aerospace Engineering, Jilin University, Changchun 130012, China; 3 School of Optoelectronic Engineering, Changchun University of Science and Technology, Changchun 130012, China; 15500457539@163.com (J.H.); shf@cust.edu.cn (H.S.)

**Keywords:** intelligent transportation system, vehicle re-identification, multi-feature fusion, vehicle detector, road traffic monitoring

## Abstract

Vehicle re-identification holds great significance for intelligent transportation and public safety. Extracting vehicle recognition information from multi-view vehicle images has become one of the challenging problems in the field of vehicle recognition. Most recent methods employ a single network extraction structure, either a single global or local measure. However, for vehicle images with high intra-class variance and low inter-class variance, exploring globally invariant features and discriminative local details is necessary. In this paper, we propose a Feature Fusion Network (GLFNet) that combines global and local information. It utilizes global features to enhance the differences between vehicles and employs local features to compactly represent vehicles of the same type. This enables the model to learn features with a large inter-class distance and small intra-class distance, significantly improving the model’s generalization ability. Experiments show that the proposed method is competitive with other advanced algorithms on three mainstream road traffic surveillance vehicle re-identification benchmark datasets.

## 1. Introduction

With the continuous advancement of computer vision technology, the field of road traffic is rapidly progressing towards informatization, efficiency, safety, and intelligence [1,2,3]. In surveillance videos, vehicles, as crucial targets, have garnered widespread attention in computer vision, encompassing tasks such as recognition [4,5], detection [6], and classification [7]. The primary objective of vehicle re-identification (Re-ID) [8,9,10,11,12] is to accurately identify the same vehicle corresponding to a given detected vehicle in surveillance videos across different scenarios or time periods. Despite the adoption of deep learning networks by many researchers in recent years to extract vehicle features [13,14] and accomplish vehicle re-identification through feature matching, challenges persist in recognition accuracy due to variations in camera heights and angles.

In the realm of vehicle re-identification, visual appearance serves as the key means of distinguishing vehicles, rather than relying on license plate information. While license plates [15] are among the most prominent features of vehicles, they may exhibit ambiguous and unreliable characteristics in certain cases. Consequently, existing research often opts for visual appearance-based methods to address challenges in vehicle re-identification tasks.

Crucial for distinguishing different vehicles are their visual details. Reference [16] posits that vehicle images with smaller spatial and temporal distances between them are more likely to belong to the same vehicle. Literature [17] identifies vehicles by reducing the distance between the same vehicle images and expanding the distance between different vehicle images. To measure distance effectively, literature [18] predominantly employs metric learning methods, directly embedding original images into Euclidean space to determine the similarity score between two vehicles. While these methods have achieved significant success in vehicle re-identification, they may be prone to confusion in the presence of subtle differences between vehicles. Table 1 summarizes representative works in recent years on vehicle re-identification.

Existing methods primarily concentrate on learning global feature representations from entire vehicle images, overlooking small and distinctive regions specific to particular vehicles. Notably, when the viewpoint or shooting angle changes, features extracted from one angle may not be applicable to another, leading to recognition failure. In such cases, these distinctive regions become crucial for vehicle re-recognition. CovaMNet [25] and DN4 [26] utilize deep local descriptors to describe the distribution of features for each category. While these methods can extract rich features, they operate only on individual support categories, lacking the ability to leverage contextual information of the entire task to generate task-specific features. In general, the importance of different parts varies dynamically across different tasks. In our local module design, we treat specific target vehicle images with the class context as a whole, thereby focusing more on the crucial aspects of the task. This approach helps avoid excessive attention to unimportant details, allowing samples within the same category to be more tightly mapped into a smaller feature space.

This paper introduces a Feature Fusion Network (GLFNet) that combines global and local information, leveraging globally invariant features and locally rich feature details. We fuse global (Global) and local (Local) information extracted by the feature extraction network using an additive representation layer. By optimizing the global structure of the vehicle detection network, we enhance differences between vehicles, improving the model performance. Simultaneously, by optimizing local information through distance measurement, we compact features of the same vehicle, reducing intra-class gaps. This framework enhances global and local relationships between different vehicle types, further improving vehicle recognition accuracy. Extensive experiments demonstrate the performance of the proposed GLFNet that combines global and local information. Our contributions are evident in the following three aspects:

(1) Introduction of a novel vehicle re-recognition framework that integrates global and local features to obtain vehicle local image feature information during the training process.

(2) Through the fusion of global and local features, the establishment of explicit key information between various parts of the vehicle object and implicit capture of discriminative local details.

(3) Comprehensive experiments on three mainstream vehicle re-identification datasets demonstrating the effectiveness of the proposed method, with experimental results indicating high accuracy.

## 2. Related Work

In this section, we provide a brief overview of relevant research in the field of vehicle re-identification. Previous methods aimed at achieving reliable vehicle re-identification have predominantly focused on leveraging visual cues, such as vehicle perspective or direction, to extract discriminative feature representations. However, the diverse angles at which cameras capture vehicles can lead to significant differences in images of the same vehicle from different directions, posing a challenge for identifying vehicles with similar orientations.

Vehicle Re-Identification: A novel strategy proposed in the literature [27] combines visual detection and feature-based search for vehicles in crowded surveillance environments. This approach begins by classifying vehicles based on their unique characteristics, followed by searching for vehicles with similar features in the dataset. Another work [28] delves into the importance of visual attributes of vehicles and introduces an innovative direction-invariant feature embedding module. This module facilitates the accurate capture and expression of direction information in vehicle images. In [29], a space–time-based approach is proposed, suggesting a visualized space–time path through innovative methods. This method displays the motion path of vehicles in space–time through graphical visualization, aiding in a deeper understanding and analysis of the dynamic behavior of vehicles. Adversarial learning methods are employed in [30] to generate new cross-view examples, enhancing learning and generalization abilities through competitive elements introduced during training. However, these methods primarily focus on learning global feature representations from entire vehicle images, overlooking small and discriminative regions that may belong to specific vehicles.

Fine-Grained Recognition: In [31], a parser is utilized to segment four views of a vehicle, and a view embedding network based on parsing is proposed to generate fine-grained feature representations. This method prioritizes the accurate understanding and description of various parts and details of the vehicle, enhancing comprehensive and accurate vehicle identification and classification. Another work, ref. [32], introduces a partial regularization method to enhance local features, strengthening feature locality and thereby improving the recognition accuracy and robustness of vehicles. A self-supervised attention method is proposed in [33] and is specifically tailored for vehicle re-recognition tasks, emphasizing the importance of self-supervised learning and attention mechanisms in extracting discriminative feature representations from vehicle images. Spatial Transformer networks are employed in [34] to locate and distinguish different regions, utilizing the self-attention mechanism of the Transformer to model the input image effectively. However, a pure Transformer is not entirely suitable for vehicle re-identification. Ref. [35] proposed a Graph Interaction Transformer (GiT) that learns discriminative local features within patches by exploring relationships between nodes. Ref. [36] introduced a Multi-Context Statistical Attention Block (MSAB) that explores semantic dependencies between channels and long-range spatial contextual dependencies between pixels through Multi-Context Channel Attention (MCA) and Statistical Spatial Attention (SSA). Ref. [37] proposed a Dual Self-Attention Module to learn different region dependencies: static self-attention for selectively enhancing semantic features and dynamic self-attention (referred to as cross-region attention) to enhance spatial awareness of local features. Ref. [38] introduced an Adaptive Interference Elimination Framework (IRF) that learns discriminative feature representations by eliminating various interferences through an attention-guided Adaptive Interference Frame Removal Module (IFRM) and attention-guided Adaptive Interference Pixel Removal Module (IPRM).

In our approach, we integrate local information while considering the global relationship. By measuring the local similarity, we compare two objects rather than two images, which is both easier and more effective. Through the fusion of global and local features, our algorithm demonstrates satisfactory results. This method comprehensively considers the overall and local information of the image, enabling more accurate object recognition and comparison.

## 3. Proposed Method

**Figure 1:**
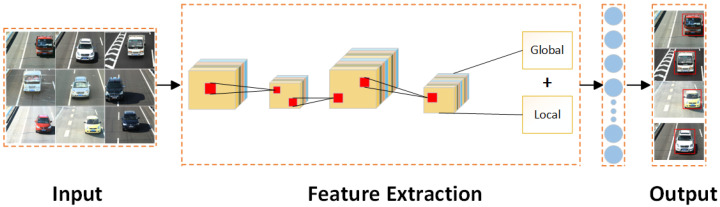
The framework diagram of the global and local information feature fusion network (GLFNet).

The Global and Local Information Feature Fusion Network (GLFNet) designed in this paper is illustrated in Figure 1. To enhance the recognition capability of vehicle image features, this study integrates the Global and Local information extracted by the feature extraction network using a summation representation layer. It utilizes the combined Global and Local information to strengthen the relationships between different vehicle models. To further improve the recognition accuracy of vehicles, this paper introduces and employs the SIOU [39] and MSE loss functions in the overall loss function. Each component will be detailed in the following sections.

### 3.1. Feature Extraction

The feature extraction network employed in this article consists of two components: an enhanced YOLOv5 network responsible for extracting global information pertaining to vehicle models, and a ResNet [40] network designed to enhance local information.

#### 3.1.1. Global Network-YOLOv5 Detection Algorithm Improved

This article proposes three enhancements to the YOLOv5 algorithm to optimize the vehicle detection network structure, amplify differences between vehicles, and enhance model performance. Firstly, an attention mechanism module (CBAM [41]) is integrated into the backbone of YOLOv5 to enhance model performance. Secondly, the SIOU loss function is applied to filter the prediction boxes, effectively addressing the issue of unstable target box regression. Lastly, DIoU-NMS [42] is employed in the place of non-maximum suppression to mitigate the loss of prediction boxes due to vehicle overlap, thereby improving detection accuracy. These three enhancements aim to optimize the YOLOv5 algorithm and enhance the accuracy and stability of vehicle detection.

(1) Backbone network

**Figure 2:**
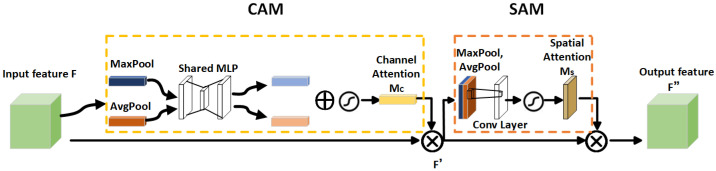
The CBAM algorithm framework takes a feature map F as the input, sequentially infers the feature map along the channel and spatial dimensions, and then adaptively optimizes it to obtain the enhanced feature map F″.

**Figure 3:**
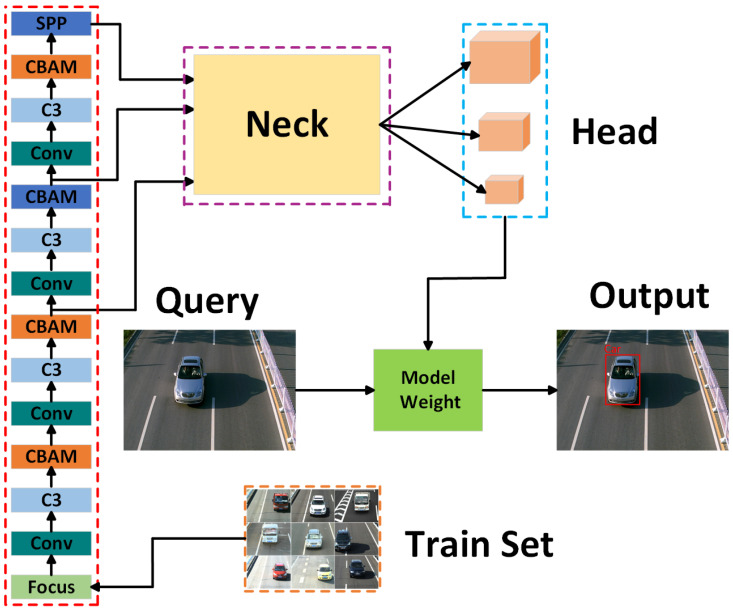
Improved backbone network structure framework of YOLOv5.

YOLOv5 is a user-friendly model known for its ease of achieving object detection. Its smaller model size and faster detection speed make it convenient for deployment on mobile devices. In this article, YOLOv5s is utilized. Upon examining the benchmark YOLOv5 network model, it was observed that it lacks an attention bias during feature extraction and employs the same weighting method for features of different importance levels. To address this, the article proposes embedding the CBAM (Attention Mechanism Module) into the four bottleneck residual block convolutions in the feature fusion region of the YOLOv5 backbone network. This adaptation enables the network to dynamically extract key features of vehicles during detection and prioritizes attention on interesting targets, ultimately improving detection accuracy. The CBAM (Convolutional Block Attention Module) is an attention mechanism module designed to enhance the feature representation ability of deep neural networks in spatial and channel dimensions. It comprises two essential components: a channel attention module and a spatial attention module. The channel attention module calculates the importance of each channel to better differentiate features in different channels. Meanwhile, the spatial attention module calculates the importance of each pixel in space to better capture the spatial structure in the image. The CBAM module can be integrated into various layers of convolutional neural networks, aiding the network in capturing important features more effectively (as illustrated in Figure 2). The backbone network structure framework of the improved YOLOv5 is shown in Figure 3.

The working process of CBAM is to input features $F\in R^{C*H*W}$ , then perform a one-dimensional convolution in the channel attention module $M_{c}\in R^{C*1*1}$ , multiply the convolution result with the original feature map, take the CAM output as the input, perform two-dimensional convolution in the spatial attention module $M_{s}\in R^{1*H*W}$ , and then multiply the output result with the original feature map.

(1) $$F^{\prime }=M_{C}\left(F\right)⊗F,$$

(2) $$F^{\prime \prime }=M_{s}\left(F^{\prime }\right)⊗F^{\prime }$$

where $M_{c}$ is a one-dimensional channel map generated from the feature map F through CAM; $M_{s}$ is a two-dimensional spatial map generated from the feature map $F^{\prime }$ through SAM; ⨂ represents an element-wise multiplication.

(2) Target detection loss function

**Figure 4:**
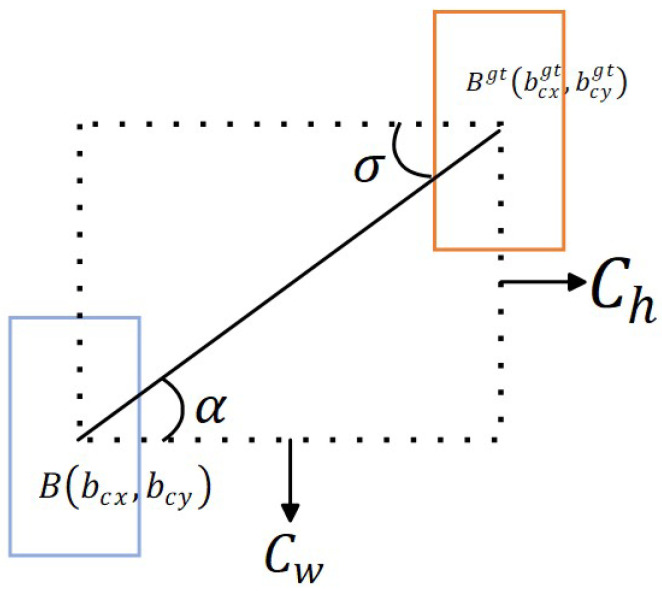
Schematic diagram of SIoU loss function.

In YOLOv5, the Generalized Intersection-over-Union (GIoU [43]) is adopted as the loss function, a bounding box prediction loss calculation method based on Intersection-over-Union (IoU). Compared with IoU, GIoU not only focuses on the overlapping area but also pays attention to other non-overlapping areas, providing a more accurate reflection of the intersection of two bounding boxes in the enclosed area. However, calculating the minimum circumscribed rectangles for each predicted and true bounding box may impose certain limitations on computation and convergence speed. Additionally, when two predicted bounding boxes completely overlap, GIoU cannot reflect the actual situation and degenerates to IoU. This paper introduces the Spatial Intersection-over-Union (SIoU) loss function for target box regression prediction. The SIoU loss function (as shown in Figure 4) more accurately evaluates the prediction results of the target box regression by comprehensively considering three aspects: angle, distance, and shape. This method effectively guides the learning process of the model, improving the prediction ability and performance.

Where *B* and $B^{g_{t}}$ represent the center points of the predicted and ground-truth boxes, respectively. $C_{W}$ and $C_{h}$ represent the differences between *B* and $B^{g_{t}}$ in the horizontal and vertical coordinates. α represents the angle between the line connecting *B* and $B^{g_{t}}$ and the horizontal line. $\left(b_{c_{x}},b_{c_{y}}\right)$ represents the center coordinates of the predicted box, $\left(b_{c_{x}}^{g_{t}},b_{c_{y}}^{g_{t}}\right)$ represents the center coordinates of the ground-truth box, and σ represents the distance between the center points of the ground-truth and predicted boxes.

The angle loss ∧ is expressed as:

(3) $$\wedge =1-2*\sin^{2}{\left(\arcsin\left(x\right)-\frac{\pi }{4}\right)}^{\leftarrow }$$

where:

(4) $$x=\frac{c_{h}}{\sigma }=sin\left(\alpha \right)$$

Redefine the distance loss Δ based on the defined angle loss:

(5) $$\Delta =\sum_{t=x,y}\left(1-e^{-\gamma \rho_{t}}\right)$$

(6) $$\rho_{x}={\left(\frac{b_{cx}^{gt}-b_{cx}}{C_{w}}\right)}^{2}$$

(7) $$\rho_{y}={\left(\frac{b_{cy}^{gt}-b_{cy}}{c_{h}}\right)}^{2}$$

(8) γ=2−Λ

where α approaches 0, Δ becomes smaller, and when α approaches π/4 , Δ becomes larger.

Shape loss Ω :

(9) $$\Omega =\sum_{t=w,h}{\left(1-e^{-wt}\right)}^{\theta }$$

where:

(10) $$w_{w}=\frac{w-w^{gt}}{max(w,w^{gt})}$$

(11) $$w_{w}=\frac{h-h^{gt}}{max(h,h^{gt})}$$

In the formula, *w* and *h* are the width and height of the true box, and $w^{g_{t}}$ and $h^{g_{t}}$ are the width and height of the predicted box.

The SIoU loss function is:

(12) $$L_{\mathrm{Global}}=1-lOU+\frac{\Delta +\Omega }{2}$$

**Figure 5:**
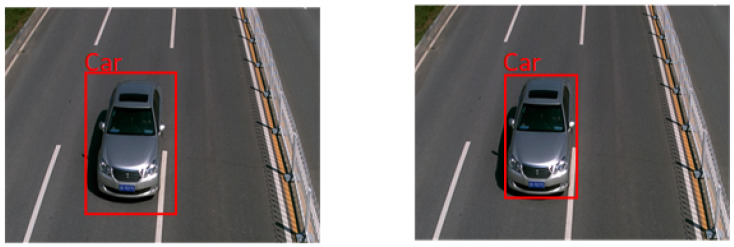
The left is the detection result of YOLOv5 using GIoU loss function, and the right is the detection result optimized using SIoU loss function.

To further illustrate the superiority of the SIoU loss function, we enhanced YOLOv5 by incorporating the SIoU loss function into the model. In comparison with the original model, the enhanced model demonstrated an improved performance in detecting prediction boxes. As depicted in Figure 5, it is evident that the position and size of the prediction box optimized using the SIoU loss function are closer to the true box. This suggests that the enhanced model can more effectively adjust the position of the bounding box, thereby mitigating the impact of boundary errors during training and enhancing the accuracy of target localization.

(3) NMS phase

In the NMS stage, for each category of detection boxes, it calculates the intersection-over-union (IOU) between the highest-scoring detection box and other detection boxes, comparing this value with a preset threshold. If the IOU exceeds the threshold, the detection box is filtered out. However, when the center points of two detection boxes are closer, the probability that they belong to the same object is greater, indicating a redundant detection box. This article introduces DIOU as a metric for NMS evaluation. Compared to traditional IOU, DIOU-NMS not only considers IOU but also takes into account the distance between the center points of two boxes. If the IOU between two boxes is relatively large but the distance between them is also large, it may be considered that these are boxes of two separate objects and will not be filtered out. This can improve the accuracy of object detection, as shown below:

(13) $$S_{i}=\left\{\begin{array}{c} S_{i},I_{IoU}-R_{DIoU}\left(M,B_{i}\right)<\varepsilon \\ 0,I_{IoU}-R_{DIoU}\left(M,B_{i}\right)\geq \varepsilon \end{array}\right.$$

where *M* represents the candidate box with high confidence, $B_{i}$ represents the process of traversing each box and comparing it with the box with high confidence, $S_{i}$ represents the score of the target classification, and ε is the preset threshold.

(14) $$R_{DIoU}=\frac{\rho^{2}\left(b,b^{gt}\right)}{c^{2}}$$

where *c* represents the longest diagonal distance between two detection boxes, and $\left(b,b^{g_{t}}\right)$ represents the distance between the center points of the two detection boxes.

#### 3.1.2. Local Network-ResNet50 Improved

(1) Network architecture

**Figure 6:**
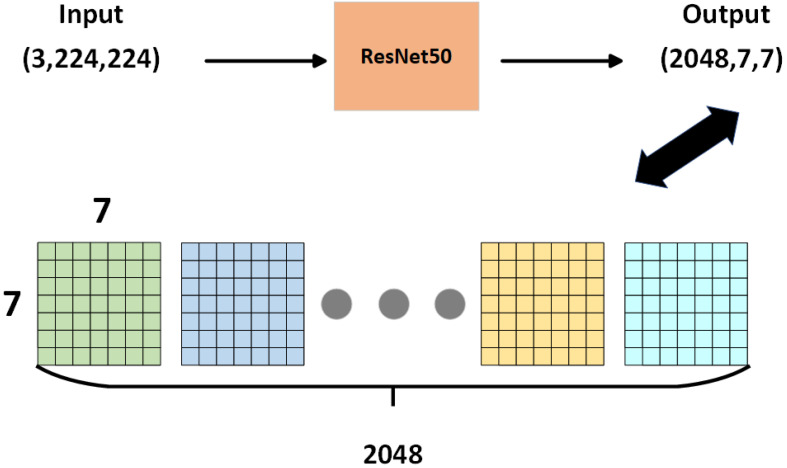
ResNet50 Network structure.

**Figure 7:**
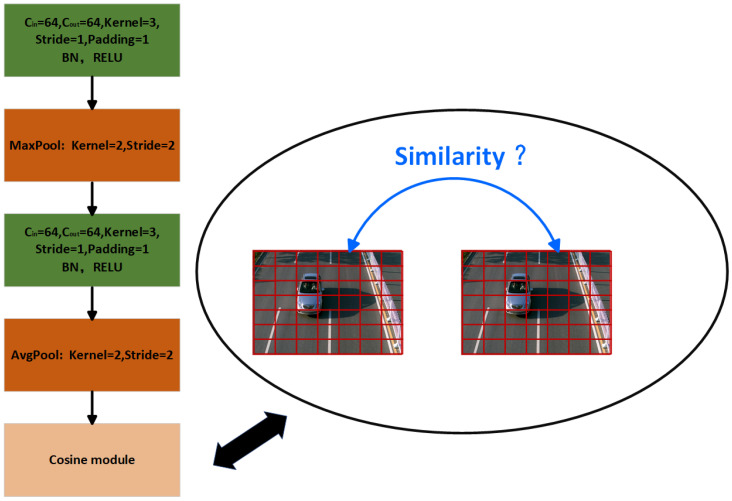
Local information network structure diagram.

This article selects the ResNet-50 network, which contains multiple residual structure units, to extract the local information of the vehicle, making the information of the same vehicle type more compact. When a 224 × 224 pixel color image is input to the network, the ResNet-50 network generates a feature map (as shown in Figure 6) with dimensions (2048, 7, 7). These feature maps then undergo local information judgment (as shown in Figure 7). The local information network structure consists of two convolutional blocks and a cosine similarity measurement layer [44]. Each convolutional block comprises 64 filters of 3 × 3 convolutions, followed by batch normalization, ReLU nonlinear activation function, and a pooling layer. The padding of each block is 1. After the first convolutional block, there is a 2 × 2 maximum pooling, and after the second convolutional block, there is a 2 × 2 average pooling. A cosine module is used to calculate the cosine distance between the query sample and the target sample for recognition. It is represented as:

(15) $$S\left(q,t\right)=h_{\cos}\left(h_{f}^{q}\left(f_{\theta }\left(x_{i}\right)\right),h_{f}^{t}\left(f_{\theta }\left(x_{i\cdots t-1}\right)\right)\right)$$

where $h_{f}^{q}\left(\cdot \right)$ represents the feature maps obtained from the ResNet network and sent to the convolutional block for information extraction. $x_{i}$ represents a selected query sample, while $x_{i\cdots t-1}$ represents the target sample. $h_{cos}$ represents the cosine module.

In the training samples, we randomly select a sample as the query sample $x_{i}^{q}$ , and set all other image samples that are similar to the specific target vehicle image as $x_{i}^{T}$ . Our cosine module does not need to connect the query sample with the target vehicle class feature representation but independently feeds the query image and class prototype feature maps to the convolution block. Next, we flatten these new feature maps and calculate the cosine distance between the query image and each class through a cosine similarity layer. The cosine similarity score represents the similarity between the query sample and the prototype of each class given the support sample. Our learning goal is to minimize the distance between samples of the same class while increasing the distance between samples of different classes. This enables clusters of vehicles of the same class to form in the feature space, helping to increase the inter-class distance of vehicle images and reduce the intra-class distance of vehicle images. In this way, our model can learn more fully, effectively, and robustly.

(2) Local loss

Using MSE Loss as the loss function for the local network:

(16) $$L_{Local}=\sum_{i}^{N}{\left(y^{g}-y\right)}^{2}$$

where $y^{g}$ represents the true label and *y* represents the predicted label.

### 3.2. Loss Function

After incorporating the global and local features, we obtain all the feature descriptors for the final vehicle. Therefore, the loss function for our global and local Information Feature Fusion Network (GLFNet) is:

(17) $$L_{Total}=\lambda_{1}L_{\mathrm{Global}}+\lambda_{2}L_{Local}$$

In order to adapt to various datasets, we set the loss weights for Global and Local features to $\lambda_{1}$ and $\lambda_{2}$ , respectively. To determine the most suitable parameters for each dataset, we employed the gradient descent method for optimization. This approach enables an automatic adjustment of the values of these two loss weights based on the dataset’s characteristics, thereby enhancing the performance of our model in the target detection task.

## 4. Performance Comparison

### 4.1. Experimental Data Set

In this section, we assess the proposed method on three widely used vehicle re-identification datasets: VeRi-776 [16], VehicleID [18], and VERI-Wild [45]. Additionally, we introduce the evaluation metrics and implementation details.

The VeRi-776 dataset comprises 51,035 images of 776 cars captured by 20 cameras. The training set includes 37,778 images of 576 cars, while the test set consists of 13,257 images of the remaining 200 cars. Within the test set, 1678 images are designated as the query set, while the remaining images form the gallery set. The dataset presents challenges due to its coverage of various perspectives, complex backgrounds, and vehicle images at different distances.

The VehicleID dataset contains 221,763 images of 26,267 vehicles. The training set includes 110,178 images from 13,134 vehicles, and the remaining images constitute the test set. The test set is further divided into three subsets: small, medium, and large, comprising images of 800, 1600, and 2400 vehicles, respectively. Notably, this dataset exclusively covers the front and back directions of vehicles, and each query image has only one matching image in the gallery.

The VERI-Wild dataset encompasses 416,314 images of 40,671 identities captured by 174 cameras over a month. The training set involves 277,797 images of 30,671 identities. The test set is also divided into three subsets, small, medium, and large, comprising images of 3000, 5000, and 10,000 identities, respectively. The dataset introduces challenges with complex backgrounds, various viewpoints, and diverse lighting and weather conditions. Moreover, multiple matching images can be present in the gallery set, adding complexity to the vehicle re-identification problem.

This paper utilizes Rank-k (Ranking Recognition Accuracy) and mAP (mean Average Precision) as evaluation metrics to assess the performance of the GLFNet network in vehicle re-identification and compares it with previously known methods. In the field of vehicle re-identification, these two metrics are widely employed as they provide a comprehensive and detailed evaluation of the algorithm’s performance across different scenarios.

The Rank-k metric measures whether the true match is included in the top k matches. Specifically, if the true match is within the top k matches, the Rank-k value is 1; otherwise, it is 0. This metric reflects the algorithm’s performance at different precision levels, as different k values can be considered. In practical applications, an appropriate k value can be selected to meet specific requirements in different scenarios.

mAP is the area under the Precision-Recall curve and serves as a comprehensive metric for evaluating system performance. It considers all possible thresholds, offering a more comprehensive understanding of the overall system performance. mAP’s performance at different precision levels is particularly useful, allowing for a comparison of the overall performance of the GLFNet network with other methods.

### 4.2. Experimental Detail

We implemented the proposed model using the PyTorch deep learning framework. During both training and testing, we adjusted the input image size to 224 × 224. Additionally, we set the batch size to 64 and determined the number of images belonging to a category in the batch based on the properties of different datasets. For the VeRi-776 dataset, we randomly selected 16 images of each vehicle identity in a batch. For the VehicleID and VERI-Wild datasets, each category had four images in a batch.

The proposed method underwent 60 training cycles based on a Global and Local Information Feature Fusion Network (GLFNet), with each training cycle consisting of 400 iterations. We employed the Adam optimizer, initializing the learning rate to 0.01. Every 20 training cycles, the learning rate was decayed by a factor of 10 until it reached 0.0001.

Throughout the training process, we applied data augmentation techniques, including rotation transformation, noise perturbation, and color jittering, to preprocess the dataset. These methods aimed to reduce irrelevant features in the dataset and enhance the overall performance of the network model. Specifically, we rotated the original image at four angles: −20°, −10°, 10°, and 20°. Additionally, we introduced Gaussian noise with a mean of 0 and a variance of 0.01, along with salt and pepper noise at a density of 0.02. Finally, we applied a histogram equalization to enhance the image’s contrast.

### 4.3. Evaluation on VeRi-776

As shown in Table 2, the proposed method outperforms other comparative methods in terms of mAP, rank1, and rank5, achieving the highest performance metrics. Specifically, the proposed method attains an mAP value of 86.49%, surpassing the highest mAP value of 79.65% observed among other methods. Regarding rank1 and rank5, the proposed method also achieves high accuracy rates of 94.43% and 98.60%, respectively, significantly surpassing the performance of other methods.

These results demonstrate that the proposed method holds significant advantages in the task of vehicle re-identification, effectively enhancing accuracy and performance. This is attributed to the adoption of novel feature extraction methods and effective feature fusion strategies, which better capture the appearance characteristics and behavioral information of vehicles, thereby improving the classification ability of the model.

### 4.4. Evaluation on VehicleID

As depicted in Table 3, various methods demonstrate distinct performances on datasets of different sizes. On small datasets, algorithms like GoogLeNet, VAMI, and TAMR exhibit stability across each subset and perform well in the small subset. EALN and PRN, on the other hand, achieve high rank1 and rank5 scores across all subsets, showcasing their robust performance in the VehicleID dataset. LOMO and FACT generally exhibit lower performance, especially in the Small and Large subsets. However, our proposed method attains the highest rank1 and rank5 accuracy across all three-sized datasets, indicating its strong generalization performance. These findings suggest that the proposed method employs effective feature extraction and classification strategies, enabling better adaptation to datasets of varying sizes and enhancing the accuracy of vehicle re-identification.

### 4.5. Evaluation on VERI-Wild

As presented in Table 4, our proposed method achieves an mAP of 83.56%, rank1 accuracy of 95.78%, and rank5 accuracy of 98.69% on the small dataset. For the medium-sized dataset, the method achieves an mAP of 78.36%, rank1 accuracy of 94.23%, and rank5 accuracy of 98.89%. On the large dataset, the method achieves an mAP of 71.56%, rank1 accuracy of 91.36%, and rank5 accuracy of 98.35%.

Comparing the proposed method with other algorithms, on small datasets, the proposed method outperforms GoogLeNet by nearly 59.29% in mAP, Triplet by nearly 67.87%, CCL by nearly 61.06%, HDC by nearly 54.42%, GSTE by nearly 52.14%, FDA-Net by nearly 48.45%, AAVER by nearly 21.21%, and SAVER by nearly 2.64%. On medium-sized datasets, the proposed method surpasses GoogLeNet by nearly 54.21% in mAP, Triplet by nearly 65.02%, CCL by nearly 59.08%, HDC by nearly 53.6%, GSTE by nearly 52.18%, FDA-Net by nearly 48.56%, AAVER by nearly 24.8%, and SAVER by nearly 3.01%. On large datasets, the proposed method exceeds GoogLeNet by nearly 50.03% in mAP, Triplet by nearly 61.63%, CCL by nearly 56.75%, HDC by nearly 53.26%, GSTE by nearly 52.06%, FDA-Net by nearly 48.78%, AAVER by nearly 29.94%, and SAVER by nearly 3.78%. These results indicate that our method exhibits a strong generalization performance and robustness, effectively handling complex scenarios and various vehicle types.

## 5. Ablation Experiment

As presented in Table 5, the ablation experiments on vehicle recognition suggest that utilizing either the YOLOv5 detection algorithm improvement (G) or the ResNet50 improvement (L) alone can enhance the performance of vehicle recognition to a certain extent. However, when combining these two improvements (YOLO+G+L), a significant improvement in performance is observed.

VeRi-776 Dataset:

YOLO+G achieves a performance of 87.43% and 92.60% on rank1 and rank5, respectively, with an mAP of 75.49. These results are obtained by improving the YOLOv5 detection algorithm.

YOLO+L shows a slightly lower performance on rank1 (83.43%) and rank5 (90.60%), but with a slightly higher mAP of 78.49 compared to YOLO+G.

YOLO+G+L significantly improves the performance on rank1 (94.43%) and rank5 (98.60%), with a notable increase in mAP to 86.49. This indicates that the GLFNet network, considering both global and local information, achieves the best performance on VeRi-776.

VehicleID Dataset:

On the VehicleID dataset, YOLO+G+L achieves a performance of 77.89% on rank1 and 91.65% on rank5, with an mAP of 82.70. This demonstrates the good performance of GLFNet on this dataset.

YOLO+G and YOLO+L, comparatively, exhibit a lower performance on rank1 and rank5, while GLFNet shows significantly better overall performance.

VERI-Wild Dataset:

On VERI-Wild, YOLO+G+L achieves a performance of 91.36% on rank1 and 98.35% on rank5, with an mAP of 71.56. This indicates that GLFNet maintains a strong performance even when faced with a more challenging dataset.

YOLO+G and YOLO+L, comparatively, demonstrate relatively lower performance on this dataset, while GLFNet exhibits the best overall performance.

In summary, GLFNet consistently demonstrates a superior performance across all three datasets, particularly when considering both global and local information. This emphasizes the excellent performance of GLFNet in the task of vehicle re-identification. These findings suggest that the combination of improvements in the YOLOv5 detection algorithm and the ResNet50 model can mutually reinforce each other, resulting in superior vehicle recognition performance. While each method individually shows improvement, their synergy leads to a significant boost in performance, possibly because these two enhancements are optimized for different aspects of vehicle recognition tasks that collectively improve the overall performance.

## 6. Case Study

**Figure 8:**
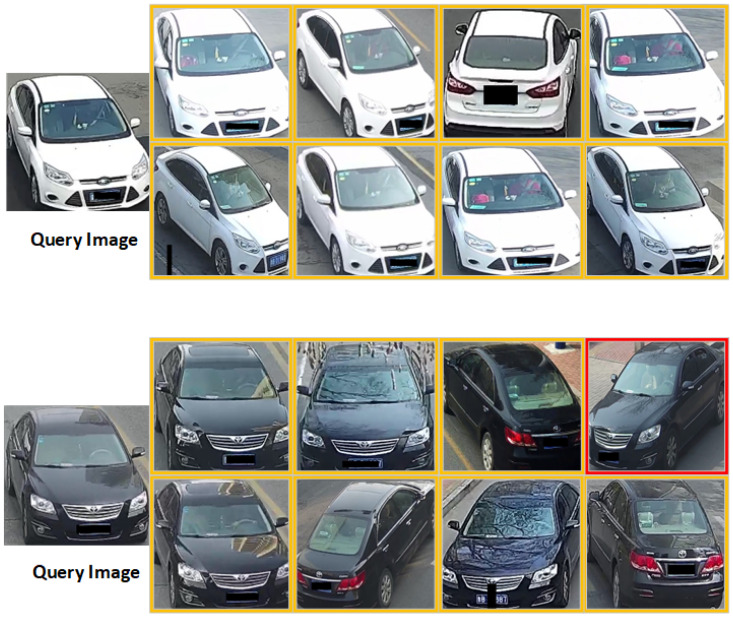
Visualization of the global and local information feature fusion network (GLFNet) (the left column shows the query images, with yellow boxes indicating correct re-recognition and red boxes indicating errors).

To further validate the effectiveness of our proposed GLFNet algorithm, re-identification retrieval experiments were conducted using vehicle target images from the VERI-Wild query set. Specifically, a Query Image was selected and processed using the GLFNet algorithm. Subsequently, the candidate images in the retrieval results were ranked. Among numerous candidate images, special attention was given to the top eight images, which were then presented visually. In Figure 8, the visualization illustrates the true matches at rank-8. The correct recognition results are highlighted in yellow, while incorrect identifications are marked in red. Through this visual representation, the performance of the GLFNet algorithm in the task of re-identifying vehicle target images can be intuitively assessed. The results clearly indicate that the algorithm proposed in this paper demonstrates outstanding performance in re-identification tasks.

## 7. Conclusions

This paper introduces a feature fusion network (GLFNet) that integrates global and local information, effectively leveraging globally invariant features and locally detailed information. By optimizing the global structure of the vehicle detection network, distinctions between vehicles are amplified. Simultaneously, through the local information optimization via a distance measurement, features of the same vehicle type are compacted, thereby reducing intra-class gaps. Extensive experiments validate the performance of the proposed feature fusion network (GLFNet) that combines global and local information. In the next phase, our focus will be on enhancing the correlation between captured images. To broaden its application scope, we plan to deploy the algorithm in a real-time video vehicle retrieval, making it more suitable for practical monitoring applications. Through these enhancements, our goal is to offer users more accurate and efficient vehicle retrieval services.

## Figures and Tables

**Table 1:** Related Work on Vehicle Reidentification.

Reference	Method Type	Advantage	Limitation
[19,20]	Deep learning	High recognition accuracy	High cost; poor interpretability
[21,22]	Spatiotemporal information	Works well for hard samples	Additional complex spatiotemporal labels are required
[16,17]	Metrics learning	High recognition accuracy	High cost
[23,24]	Multidimensional information based	Sensitivity to the special appearance of vehicles	Susceptible to changes in viewpoints and illuminations

**Table 2:** Comparison of Different Vehicle Re-identification Algorithms on the VeRi-776 Dataset. Bold black text indicates the highest value in each column.

Methods	mAP	rank1	rank5
LOMO [46]	9.62	25.42	46.51
DGD [47]	18.35	49.84	67.45
GoogLeNet [48]	17.81	52.12	66.78
FACT [16]	18.73	51.85	67.16
XVGAN [49]	24.65	60.20	77.03
OIFE [28]	48.21	65.92	87.66
FDA-Net [45]	55.60	84.27	91.41
GSTE [50]	59.43	92.21	96.25
SAVER [33]	79.65	93.42	95.66
VAAG [51]	65.23	91.35	94.76
NVSL [52]	67.58	91.52	94.83
APANet [53]	78.61	93.35	96.49
**Ours**	**86.49**	**94.43**	**98.60**

**Table 3:** Comparison of Different Vehicle Re-identification Algorithms on the VehicleID Dataset. Bold black font indicates the highest value in each column.

Methods	Small		Medium		Large	
	rank1	rank5	rank1	rank5	rank1	rank5
LOMO [46]	19.84	32.21	18.94	29.12	15.36	25.23
GoogLeNet [48]	47.52	67.23	43.56	63.82	38.23	59.63
VAMI [54]	63.56	83.21	52.86	75.13	47.36	70.32
TAMR [55]	66.89	79.75	62.93	76.86	59.35	73.68
FACT [16]	49.53	68.01	44.56	64.59	40.24	60.35
EALN [30]	75.56	88.23	71.83	83.92	68.92	81.46
PRN [32]	78.65	92.35	75.02	88.35	74.23	86.42
RAM [56]	75.35	91.56	72.36	87.60	67.26	84.56
KPEV [22]	72.23	87.41	68.93	84.52	63.92	78.29
Transfer [57]	52.76	67.29	47.65	63.83	43.87	62.43
TCPM [58]	81.95	94.81	77.66	92.91	73.19	90.35
**Ours**	**83.56**	**96.23**	**79.56**	**93.66**	**77.89**	**91.65**

**Table 4:** Comparison of Different Vehicle Re-identification Algorithms on the VERI-Wild Dataset. Bold black font indicates the highest value in each column.

Methods	Small			Medium			Large		
	**mAP**	**rank1**	**rank5**	**mAP**	**rank1**	**rank5**	**mAP**	**rank1**	**rank5**
GoogLeNet [48]	24.27	57.16	75.13	24.15	53.16	71.13	21.53	44.61	63.55
Triplet [59]	15.69	44.67	63.33	13.34	40.34	58.98	9.93	33.46	51.36
CCL [18]	22.50	56.96	75.36	19.28	46.16	69.88	14.81	37.94	59.89
HDC [60]	29.14	57.23	78.93	24.76	49.64	72.28	18.30	43.98	64.95
GSTE [50]	31.42	60.48	80.13	26.18	52.13	74.98	19.50	45.36	66.53
FDA-Net [45]	35.11	64.23	82.95	29.80	57.83	78.64	22.78	49.43	70.48
AAVER [61]	62.35	75.86	92.76	53.56	68.23	88.79	41.62	58.63	81.65
SAVER [33]	80.92	92.56	96.56	75.35	90.56	95.48	67.78	85.65	95.86
**Ours**	**83.56**	**95.78**	**98.69**	**78.36**	**94.23**	**98.89**	**71.56**	**91.36**	**98.35**

**Table 5:** Ablation experiments on three datasets (Bold black font indicates the highest value in each column). G: YOLOv5 detection algorithm improvement; L: ResNet50 improvement.

Methods	VeRi-776			VehicleID			VERI-Wild		
	**rank1**	**rank5**	**mAP**	**Large**			**Large**		
				**rank1**	**rank5**	**mAP**	**rank1**	**rank5**	**mAP**
YOLO+G	87.43	92.60	75.49	70.36	84.65	75.62	82.45	92.51	65.28
YOLO+L	83.43	90.60	78.49	73.88	86.65	72.95	81.27	91.78	69.84
YOLO+G+L	**94.43**	**98.60**	**86.49**	**77.89**	**91.65**	**82.70**	**91.36**	**98.35**	**71.56**

## Data Availability

Data are contained within the article.
